# Aging, Transition, and Estimating the Global Burden of Disease

**DOI:** 10.1371/journal.pone.0020264

**Published:** 2011-05-24

**Authors:** Benjamin J. Seligman, Mark R. Cullen, Ralph I. Horwitz

**Affiliations:** 1 Stanford University School of Medicine, Palo Alto, California, United States of America; 2 Department of Medicine, Stanford University School of Medicine, Palo Alto, California, United States of America; 3 GlaxoSmithKline, Philadelphia, Pennsylvania, United States of America; University of Zaragoza, Spain

## Abstract

**Background:**

The World Health Organization's Global Burden of Disease (GBD) reports are an important tool for global health policy makers, however the accuracy of estimates for countries undergoing an epidemiologic transition is unclear. We attempted to validate the life table model used to generate estimates for all-cause mortality in developing countries.

**Methods and Results:**

Data were obtained for males and females from the Human Mortality Database for all countries with available data every ten years from 1900 to 2000. These provided inputs for the GBD life table model and served as comparison observed data. Above age sixty model estimates of survival for both sexes differed substantially from those observed. Prior to the year 1960 for males and 1930 for females, estimated survival tended to be greater than observed; following 1960 for both males and females estimated survival tended to be less than observed. Viewing observed and estimated survival separately, observed survival past sixty increased over the years considered. For males, the increase was from a mean (sd) probability of 0.22 (0.06) to 0.46 (0.1). For females, the increase was from 0.26 (0.06) to 0.65 (0.08). By contrast, estimated survival past sixty decreased over the same period. Among males, estimated survival probability declined from 0.54 (0.2) to 0.09 (0.06). Among females, the decline was from 0.36 (0.12) to 0.15 (0.08).

**Conclusions:**

These results show that the GBD mortality model did not accurately estimate survival at older ages as developed countries transitioned in the twentieth century and may be similarly flawed in developing countries now undergoing transition. Estimates of the size of older-age populations and their attributable disease burden should be reconsidered.

## Introduction

Estimating the mortality and morbidity burden of diseases worldwide is an important part of setting meaningful global health priorities. Since the 1990s, Global Burden of Disease (GBD) series of reports estimate rates of mortality and morbidity for countries that lack systems of vital registration or disease registries. These reports [1]–[3] and related papers [4], [5], which are currently produced by the World Health Organization, are frequently referenced and cited by governments and NGOs when setting their priorities for funding in health.

It is straightforward to produce estimates for the numbers of deaths by cause for countries that maintain vital registration systems with complete, national coverage. For many developing countries, which lack such systems, the process requires mathematical modeling to generate estimates. For the GBD reports, a two-step process was used: first, a “mortality envelope” of deaths by age and sex was estimated using a demographic model [6] to produce mortality rates and United Nations population data to estimate numbers of deaths. A second model then estimated the proportion of deaths by cause by age for each sex [7]. This process was required for 92 out of 192 WHO member states in the last full report, published in 2006 [2].

Of concern in these models is their development and validation using data primarily from developed countries, despite being intended for estimation of mortality in developing countries. Complicating this and potentially limiting options for straightforward correction is the epidemiologic transition. This is the phenomena in which countries, as they develop socially and economically, move toward lower, if still significant, burdens of communicable disease and perinatal mortality and greater burdens of non-communicable chronic diseases (NCDs). While the nature of the transition is well-described among now-developed countries, it is unclear if their experience of transition, which occurred at higher levels of economic development and at a slower pace, can represent that of developing countries today.

If data were available from suitable sources, the GBD model could be validated for the very populations in the developing world where its model estimates have their greatest application. Since such data are not available, we chose instead to test the model's performance for countries in the developed world with reliable data as they underwent transition over the course of the 20^th^ century. We presumed that assessment of the GBD model against a database of vital registration data on mortality for developed countries would illustrate performance characteristics of the model and suggest potential limitations in application to developing countries currently transitioning.

## Methods

The life table is a tool in demography that describes the mortality experience of a synthetic population comprised of a series of cross-sectional groups of individuals. Thus, rather than following a cohort of people through time to measure their survival, the life table presents current mortality rates in each age group and across both sexes in an existing population. While this approach presents a generic limitation to the use of life tables as predictive tools, they are invaluable for understanding current mortality in a given population. Life tables can be used to produce hypothetical estimates, such as life expectancy and survival probabilities. In calculating survival probabilities, one asks what fraction of individuals of a given age would survive to a certain later age if existing mortality rates did not change. Life expectancy asks what mean survival would be for children born that year if current mortality rates remained constant over their lifetime. Conversely, the survival probabilities could be used to estimate the total number of deaths across all age groups in a given population, as is done in the GBD series.

For developing countries, a model life table was developed and used by the GBD authors. This model is a modified Brass logit life table system, which is described fully elsewhere [6]. Briefly, the Brass logit life table system treats life tables as linear transformations of a standard life table. A contribution of the GBD authors was to make this system more robust in situations where the relationship between the estimated and standard life tables is not linear. Their extension was developed using 1,802 life tables from the 20^th^ century, predominantly from European, North American, and Australasian countries. The formulae they used for estimating life tables are shown in Text S1.

**Figure 1:**
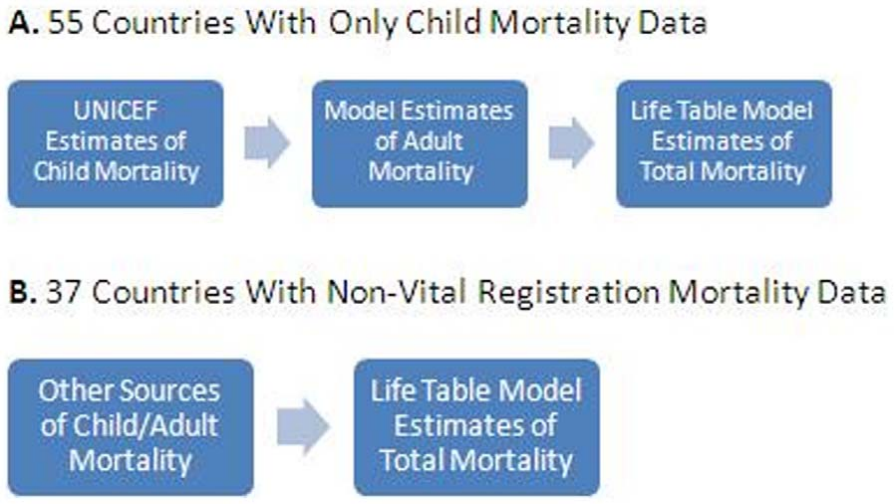
This figure outlines methods used in the GBD for estimating mortality in countries without complete vital registration data. A shows the GBD model for arriving at mortality estimates for countries with only UNICEF child mortality estimates. B shows this process for countries that have mortality data that are not from vital registries.

The model life table used in the GBD requires inputs of survival from birth to ages five and sixty. For 55 of the 92 countries that required model estimation of all-cause mortality, only UNICEF estimates for child survival to age five were available. These estimates were linked to survival to age sixty using regression techniques in order to produce survival data to ages 5 and 60 for the demographic model [8]; we do not assess this model in this paper. In the remaining 37 countries, available data, including surveys and incomplete vital registration data, were used to produce model inputs. These processes are outlined in Figure 1.

This GBD model life table uses the available data to estimate mortality up to age 85. During the development of the GBD model, the investigators validated its performance using standard techniques of split sample evaluation, separating the sample into training and test sets. These assessments confirmed the internal validity of the model. However, validation against an external population has not been published.

To assess the performance of the GBD, we used data from the Human Mortality Database (HMD) [9]. This database contains life tables for 37 countries dating back as far as the year 1751. For this study, we obtained “observed” mortality rates by using life tables for males and females for the first year of every decade beginning with 1900 for all 37 countries. For those countries where data were not available in 1900, the first year of a decade with available data was used. The Federal Republic of Germany, the German Democratic Republic, and re-unified Germany were treated separately. Scotland was analyzed separately from England and Wales and Northern Ireland was not analyzed. For France and New Zealand, national populations were used.

These life tables also provided the inputs—survival from birth to ages 5 and 60– for the above formulae to construct “estimated” life tables using the GBD model. Data were analyzed using R version 2.10.0 [10].

## Results

We first evaluated the ratio of estimated to observed survival for ages fifteen to sixty from 1900 to 2000 for all countries for all years. As expected, the GBD model “estimates” of survival closely matched the observed survival rates (data not shown). We further explored error between ages fifteen and sixty and found some underestimation of mortality rates, however this was small and did not vary in magnitude over time (data not shown). It can be shown that the model's estimated survival from birth to age five reduces to the input observed survival from birth to age five, and thus it was not evaluated.

**Figure 2:**
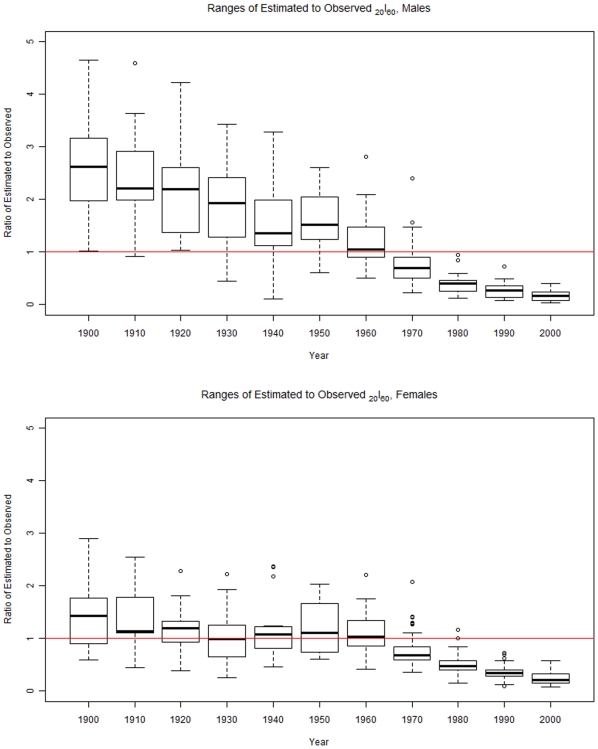
This figure shows box-and-whisker plots of the ratio of GBD model estimated survival from age 60 to 80 to the observed survival for each year in the set of life tables for males (top) and females (bottom).

Box plots showing the ratio of estimated to observed survival rates from age 60 to age 80 (*_20_l_60_*) from 1900 to 2000 are displayed in Figure 2; the year of the data is given along the x-axis and the ratio of the model-estimated to HMD-observed survival probability is given along the y-axis. As this figure shows, the GBD model substantially over-estimates survival probabilities among males in the first half of the 20^th^ century, whereas for females it performs reasonably well. However, in the latter half of the century, the model overestimates mortality for both males and females, with ever increasing deviation from observed mortality over time.

**Figure 3:**
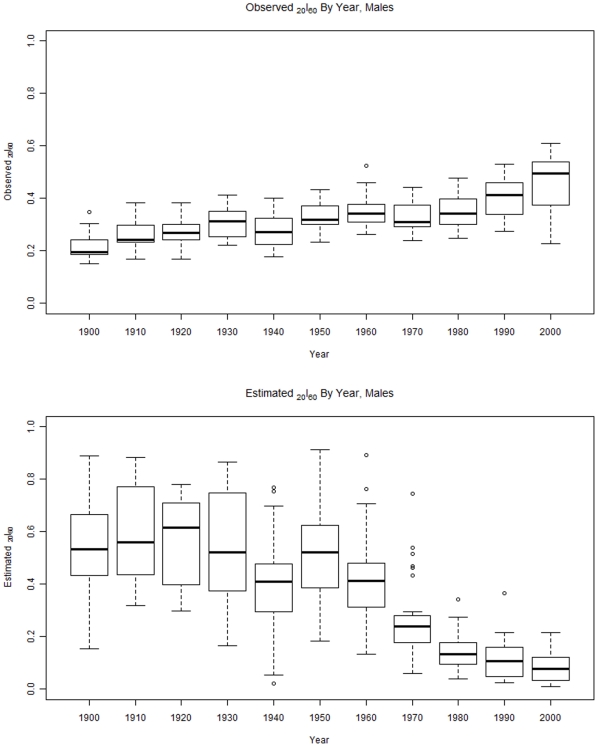
This figure shows observed survival from age 60 to age 80 for males from 1900 to 2000 (top) and the estimated survival for males in the same period (bottom).

**Figure 4:**
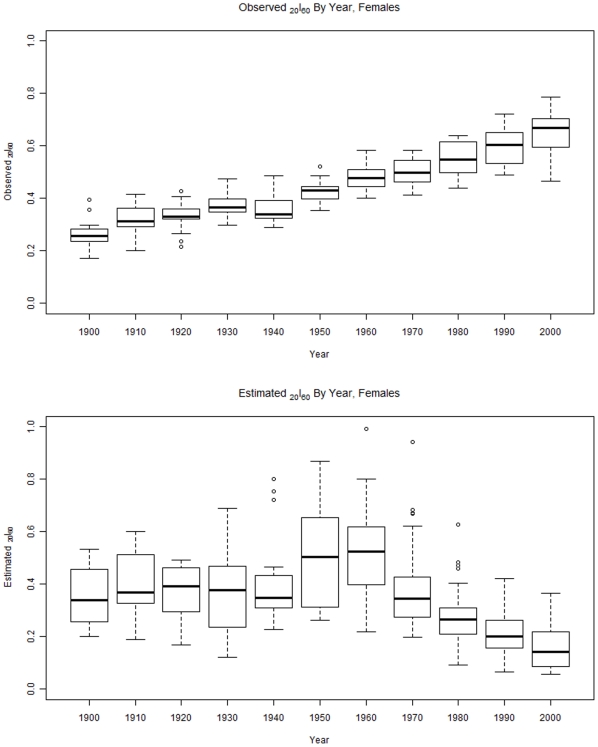
This figure shows observed survival from age 60 to age 80 for females from 1900 to 2000 (top) and the estimated survival for females in the same period (bottom).

To decompose these findings, plots of estimated and observed *_20_l_60_* were produced separately for males and females (Figures 3 and 4). These figures show that for both sexes, actual or observed survival probabilities have been *increasing* with time. For males, the increase was from a mean (sd) probability of 0.22 (0.06) to 0.46 (0.1). For females, the increase was from 0.26 (0.06) to 0.65 (0.08).

By contrast, survival probabilities estimated from the GBD model have been *decreasing* over the same period of time Among males, estimated survival probability declined from 0.54 (0.2) to 0.09 (0.06). Among females, the decline was from 0.36 (0.12) to 0.15 (0.08).

## Discussion

Our results demonstrate that, through time measured from the beginning of the 20^th^ century to the beginning of the 21^st^, the GBD model life table systematically misestimates survival from age sixty to age eighty in a sample of countries that experienced substantial economic (and other) development over that period. Before 1960, the GBD model tends to overestimate survival; after 1960, it tends to underestimate survival. This impairment in model performance holds true across the diversity of countries in the sample, which includes both Western and Soviet Bloc countries, European and Asian populations, and countries that, for at least a portion of the years sampled, were considered developing countries. Further, the model estimates a trend of increasing mortality at older ages through time, whereas the observed trend is of declining mortality at these ages.

These findings contradict the conclusions of the validation attempted by the model's developers [7]. In the paper describing this model, they used a sample of 1,802 life tables, roughly 1,000 of which were from Western countries, Japan, and Singapore between 1950 and 1998. Even with life tables from the countries and years included in the model, however, it does not accurately estimate survival at older ages except for the year 1960. This is approximately the weighted average of the years represented among 1,802 life tables.

This suggests that the model treats countries as facing a relatively static mortality pattern, specifically that of the middle of the 20^th^ century. This is likely to be inaccurate for countries transitioning today, as it was for describing the developed countries in our sample as they transitioned.

The findings reported in this paper also call into question the mortality estimates at older ages presented in the GBD reports, particularly in light of the assertion in the update published in 2008 that half of global mortality occurs after age sixty. Our data and analysis suggests that mortality at these older ages in developing countries is being over- or under-estimated, possibly substantially. Given the rapid increases in life expectancy worldwide, with the possible exception of Sub-Saharan Africa, the concern for over-estimation among the 92 countries where this model was applied would appear far more likely.

Our study was not designed to identify the specific reasons for the GBD model's poor performance. Our analysis does illustrate an important general conclusion that has far-reaching policy implications. When Western countries were experiencing the economic transitions that developing countries are experiencing now, the proportion of the population at older ages in Western nations was likely much lower. The GBD model fails to accurately estimate the growing number of older persons in developing countries at the very time when their economic resources are already strained and as they attempt to meet the healthcare needs of their populations.

An additional limitation to this study, which strongly suggesting there is substantial error in GBD estimates, is that we cannot quantify that error. In particular, we are unable to specify to what extent the model under- or over-estimates survival from age sixty to age eighty. If one believes that contemporary developing countries as a whole closely resemble developed countries in 1960 one may even argue that there is little error in this estimate, however their diversity as a group and the rapidity of their individual epidemiologic transitions make this unlikely.

Current estimates of older-age mortality in the GBD reports that are not derived from count-based data should not be accepted as valid uncritically nor as representing the actual mortality experience of those in the population over the age of sixty. Further work to improve models based to the fullest extent possible on inferences from observations in low/middle income countries, however imperfect, will be necessary to restore confidence in the mortality estimates derived from the GBD model and to ensure appropriate allocation of resources for global health.

## Supporting Information

Text S1 Briefly outlines the formulae used in the GBD model life table.
(DOC)
