# Supplementary material for: Aging, Transition, and Estimating the Global Burden of Disease
Source: PLoS One. 2011 May 24;6(5):e20264. doi: 10.1371/journal.pone.0020264 (PMC3101233; doi:10.1371/journal.pone.0020264)
Supplement: Text S1 — Briefly outlines the formulae used in the GBD model life table. (DOC) [file pone.0020264.s001.doc]

# Text S1.

The analysis of the GBD authors yielded the following formula for estimating survival to age x for population j in year i:

$$\log it(l_{xij})=\alpha_{ij}+\beta_{ij}*\log it(l_{x}^{s})-\gamma_{x}\left(1-\frac{\log it(l_{5ij})}{\log it(l_{5}^{s})}\right)-\theta_{x}\left(1-\frac{\log it(l_{60ij})}{\log it(l_{60}^{s})}\right)$$

Where $l_{x}^{s}$ is survival to age x in the standard life table and α and β are:

$$\alpha_{ij}=\frac{\log it(l_{5ij})*\log it(l_{60}^{s})-\log it(l_{5}^{s})*\log it(l_{60ij})}{\log it(l_{60}^{s})-\log it(l_{5}^{s})}$$

$$\beta_{ij}=\frac{\log it(l_{60ij})-\log it(l_{5ij})}{\log it(l_{60}^{s})-\log it(l_{5}^{s})}$$

Values for γ, θ, and $l_{x}^{s}$ were estimated empirically from their database of life tables. Thus, the only values that must be provided are survival to age five and survival to age sixty in the target population, or $l_{5ij}$and $l_{60ij}$ respectively.
